# Guidance on physical activity from cancer survivorship to pregnancy: a scoping review

**DOI:** 10.3389/fpubh.2026.1801672

**Published:** 2026-05-29

**Authors:** Anna Weronika Szablewska, Katarzyna Pietrzak, Alberto Alves, Anna Szumielewicz, Carla Malveiro, Sandra Silva-Santos, Rita Santos-Rocha

**Affiliations:** 1 Department of Obstetric and Gynaecological Nursing, Institute of Nursing and Midwifery, Medical University of Gdańsk, Gdańsk, Poland; 2 Independent Monoprofile Medical Simulation Laboratory, Institute of Nursing and Midwifery, Faculty of Health Sciences with the Institute of Maritime and Tropical Medicine, Medical University of Gdańsk, Gdańsk, Poland; 3 Research Centre in Sport Sciences, Health and Human Development (CIDESD), University of Maia, Maia, Portugal; 4 ONCOMOVE–Associação de Investigação de Cuidados de Suporte em Oncologia (AICSO), Vila Nova de Gaia, Portugal; 5 National Program for the Promotion of Physical Activity, Directorate-General of Health, Lisbon, Portugal; 6 Department of Physical Activity for Women’s Health, Gdansk University of Physical Education and Sport, Gdańsk, Poland; 7 Breast Cancer Research Program, Champalimaud Foundation, Lisbon, Portugal; 8 Exercise and Health Laboratory, CIPER, Faculty of Human Kinetics, University of Lisbon, Lisbon, Portugal; 9 Research Centre in Development and Innovation – CIDI/European Institute of Higher Education – Fafe, Fafe, Portugal; 10 SPRINT-Sport Physical Activity and Health Research & Innovation Center, Lisbon, Portugal; 11 Piaget Research Centre for Ecological Human Development, Higher School of Sport and Education, Jean Piaget Polytechnic Institute of the North, Vila Nova de Gaia, Portugal; 12 Department of Physical Activity and Health, ESDRM Sport Sciences School of Rio Maior, Santarém Polytechnic University, Rio Maior, Portugal

**Keywords:** cancer survivors, exercise, fitness, physical activity, pregnancy

## Abstract

**Background:**

Improvements in cancer survival have resulted in a growing number of women of reproductive age who plan pregnancy or become pregnant after cancer. Physical activity is widely recommended during pregnancy and across the cancer survivorship continuum; however, there is currently no existing guidance adequately addressing women who fall at the intersection of these contexts. This scoping review was aimed at mapping existing guidance on physical activity during pregnancy and cancer survivorship, and to identify gaps relevant to women planning pregnancy or who become pregnant after cancer treatment.

**Materials and methods:**

A scoping review of clinical practice guidelines, position statements and consensus documents addressing physical activity during preconception, pregnancy, postpartum or cancer survivorship was conducted. Sources were identified through database searches. Data were presented according to population (preconception, pregnancy, postpartum, cancer survivorship, pregnancy after cancer) and recommendation domains, including exercise prescription, safety, cancer treatment-related considerations, monitoring and progression, and implementation pathways.

**Results:**

A total of *N* = [34] sources met the inclusion criteria. Guidance specifically addressing the preconception period was sparse; accordingly, the pregnancy-focused corpus was dominated by recommendations for pregnancy, with more limited reference to preconception and postpartum. Pregnancy guidelines showed high concordance in recommending at least 150 min per week of moderate-intensity physical activity for women without contraindications and provided structured safety guidance. Cancer survivorship guidance consistently endorsed regular physical activity and avoidance of inactivity, often recommending similar activity doses, while more comprehensively addressing treatment-related late effects and implementation considerations. No source provided integrated or population-specific guidance for physical activity in women planning pregnancy or becoming pregnant after cancer. This absence was consistent across all recommendation domains.

**Conclusion:**

Although physical activity guidance is well established for pregnancy and cancer survivorship independently, recommendations remain siloed and do not address the needs of women navigating pregnancy after cancer. Integrated, interdisciplinary guidance is needed to support safe, individualised physical activity participation in this growing population.

**Systematic review registration:**

https://doi.org/10.17605/OSF.IO/KJVPS.

## 1 Introduction

In recent years, global trends have shown a noticeable shift toward delayed motherhood, particularly in high-income countries (1). As women increasingly postpone childbearing to later stages of reproductive life, the risk of cancer diagnoses during or prior to childbearing age grows. Epidemiological data suggest that cancers of the, e.g., breast, thyroid cancer, melanoma, cervical and Hodgkin lymphoma, are among the most common malignancies diagnosed in women of reproductive age (2–4). Thanks to advances in oncology, fertility-preserving treatment options and supportive care, more women who have survived cancer now consider pregnancy as a safe and achievable life goal (5, 6).

Despite improved survival rates, a history of cancer and its treatment,—including chemotherapy, radiotherapy and surgical interventions—may pose unique risks to both maternal and foetal health during pregnancy (6). Cancer therapies can affect ovarian reserve, uterine function and cardiovascular health, potentially influencing pregnancy outcomes such as pre-term birth, low birth weight, hypertensive disorders and even neonatal health (7). Consequently, pregnancy after cancer is increasingly viewed not only as a medical, but also a psychosocial challenge requiring holistic and evidence-based care.

Physical activity is widely recognised as an important element in maintaining overall health. Undertaking regular physical activity contributes to improved cardiovascular function, metabolic balance, mental well-being and musculoskeletal strength (8). Among cancer survivors, physical activity has been proven to reduce fatigue, enhance quality of life, improve immune function and lower the risk of cancer recurrence as well as secondary complications (9). Additionally, in the context of fertility and pregnancy, physical activity has been linked to improved reproduction, including higher chances of conception, better pregnancy tolerance, reduced obstetric complications and healthier neonatal outcomes (10).

Given the growing population of women who have survived cancer and wish to become pregnant, and the well-documented health benefits of physical activity for both general and oncology populations, it is essential to examine whether specific guidelines exist regarding exercise during pregnancy for this vulnerable-subgroup. Currently, there is a lack of standardised recommendations for physical activity tailored to the needs of pregnant women with a history of cancer. This creates uncertainty for both healthcare providers and patients when counselling about exercise and lifestyle management in this context. More specifically, it remains unclear whether existing guidance provides sufficient detail on the type, intensity, frequency, and progression of physical activity that may be appropriate for women planning pregnancy or becoming pregnant after cancer. There is also uncertainty regarding how contraindications, treatment-related late effects, obstetric risk factors, and safety monitoring should be integrated when advising this population. This lack of specificity limits the clinical usefulness of currently available guidance for women at the intersection of cancer survivorship and pregnancy.

A scoping review approach was chosen to provide an overview of the existing guidelines, expert consensus, and recommendations, regardless of study design, and to identify knowledge gaps in this emerging field.

Pregnancy after cancer represents a distinct physiological and clinical context: persistent treatment-related sequelae,—such as cardiopulmonary impairment, metabolic and endocrine dysregulation, neuromuscular limitations, skeletal fragility, lymphatic complications and chronic symptoms (e.g., fatigue or neuropathy) (11–13)—may interact with the evolving physiological demands and contraindications to pregnancy. Consequently, recommendations developed for pregnancy or cancer survivorship in isolation may not be directly transferable to women who are cancer survivors planning pregnancy or who become pregnant after treatment. To our knowledge, in no previous scoping review has it been mapped and critically described how physical activity guidance addresses (or fails to address) this combined population. Therefore, the aim of this scoping review is to systematically identify and map published guidance documents relevant to physical activity in cancer survivorship and in pregnancy, and to characterise the extent to which they provide clinically actionable recommendations for women with a history of cancer who are planning pregnancy or are pregnant. By delineating what guidance exists and where it is absent, the aim of this review is to inform clinical practice and shared decision-making, and to support future development of integrated care pathways and research priorities.

## 2 Materials and methods

The protocol for this scoping review was prospectively registered in the Open Science Framework (OSF, DOI: 10.17605/OSF.IO/KJVPS). This review was conducted in accordance with PRISMA-ScR guidelines (14). Given the exploratory nature of the research question and the anticipated heterogeneity of sources (e.g., guidelines, consensus statements, position papers), we conducted a scoping review to map available guidance and identify evidence gaps rather than evaluate intervention effectiveness. The methodological framework is based on the five-stage approach proposed by Arksey and O’Malley, later modified by Levac et al. (15, 16), and further advanced by the Joanna Briggs Institute (JBI) (17). Ethical approval was not required, as this study involved the use of previously published data.

### 2.1 Stage 1: identifying the research questions

The following research questions were addressed in this scoping review:

- What physical activity recommendations currently exist for women with a history of cancer who plan pregnancy or are pregnant?
- What are the characteristics and scope of these recommendations (e.g., type, intensity, frequency of activity)?
- What gaps exist in the current literature regarding physical activity guidelines for this population?

To structure the research questions, the PCC (Population, Concept, Context) framework was applied (Table 1).

**Table 1:** PCC framework applied to the research questions.

PCC element	Description
Population	Women with a history of cancer and women planning pregnancy or pregnant, with particular emphasis on the intersection of these populations (i.e., women planning pregnancy or pregnant after cancer)
Concept	Physical activity and exercise recommendations (type, frequency, duration and intensity)
Context	Clinical practice guidelines, consensus statements, national or international recommendations related to oncology or obstetrics

### 2.2 Stage 2: identifying relevant studies

Separate search strategies were developed for two target populations: cancer survivors and women in the context of pregnancy or pregnancy planning. Separate search strategies were intentionally developed for the two target populations in order to maximise sensitivity and to map the available guidance in each evidence corpus before examining whether any integrated recommendations existed at their intersection. A search strategy restricted only to the direct combination of both populations did not retrieve records and therefore would not have allowed meaningful mapping of the guidance streams that currently inform practice in isolation. For this reason, the review was designed to first map guidance in cancer survivorship and in pregnancy/preconception/postpartum separately, and then assess the extent to which either corpus addressed the combined population. Searches were conducted between April and November 2025 in PubMed, Scopus, Web of Science, Embase and Google Scholar. The full electronic search strategies are presented in Tables 2,3. Searches were limited to documents published in English between 2015 and 2025.

**Table 2:** Search strategy—cancer survivors.

Database	Date of search	Search string	Filters	Records retrieved
Pub Med	14 Apr. 2025–20 Nov. 2025	(“physical activity” [tiab] OR “exercise” [tiab]) AND (“cancer survivors” [MeSH] OR “cancer survivor*” [tiab] OR survivorship [tiab]) AND (guideline* [tiab] OR recommendation* [tiab] OR consensus [tiab] OR statement [tiab])	English; 2015–2025; Consensus development Conference, Guideline, Practice guideline, Systematic review, Scoping review	166
Scopus	14 Apr. 2025−20 Nov.	TITLE-ABS-KEY ((“physical activity” OR exercise) AND (“cancer survivor*” OR “oncology patient*”) AND (guideline* OR recommendation* OR consensus OR “position statement” OR “Delphi study”))	English; 2015–2025; Limited to: Medicine, Nursing, Health professions (advanced search)	1,371
Web of Science	14 Apr. 2025–20 Nov.	Advanced search → Topic: “physical activity” OR exercise (add row) And/Topic: “cancer survivor*” OR “oncology patient*” (add row) And/Topic: guideline* OR recommendation* OR consensus OR “position statement” OR “Delphi study” https://www.webofscience.com/wos/woscc/summary/982afca5-ed91-44e0-a5b5-6d8453654c67-0181dd0ebf/relevance/1	English; Publication dates: 2015-04-15 to 2025-10-18	1,643
Embase	14 Apr. 2025–20 Nov.	(‘physical activity’:ab,ti OR ‘exercise’:ab,ti) AND (‘cancer survivor*’:ab,ti OR ‘oncology patient*’:ab,ti) AND (‘guideline*’:ab,ti OR ‘recommendation*’:ab,ti OR ‘consensus’:ab,ti OR ‘Delphi study’:ab,ti)	English; 2015–2025; Consensus development, Delphi study, Clinical practice guideline, Systematic Review; Gender: women	1,904

**Table 3:** Search strategy—pregnancy/pregnancy planning.

Database	Date of search	Search string	Filters	Records retrieved
PubMed	14 Apr. 2025–20 Nov 2025	(“physical activity”[tiab] OR “exercise” [tiab]) AND (“Pregnancy” [MeSH] OR “pregnant women” [tiab] OR “pregnancy planning” [tiab] OR preconception [tiab]) AND (“guideline*” [tiab] OR “recommendation*” [tiab] OR “consensus” [tiab] OR “position statement” [tiab] OR “Delphi study” [tiab])	English; 2015–2025; Consensus development Conference, Guideline, practice Guideline, Systematic review, Scoping review	158
Scopus	14 Apr. 2025–20 Nov. 2025	TITLE-ABS-KEY ((“physical activity” OR exercise) AND (pregnancy OR “pregnant women” OR “pregnancy planning” OR preconception) AND (guideline* OR recommendation* OR consensus OR “position statement” OR “Delphi study”))	English; 2015–2025; Limited to: Medicine, Nursing, Health Professions; (advanced search)	1,859
Web of Science	14 Apr. 2025–20 Nov. 2025	Advanced search → Topic: “physical activity” OR exercise (add row) And/Topic: pregnancy OR “pregnant women” OR “pregnancy planning” OR preconception (add row) And/Topic: guideline* OR recommendation* OR consensus OR “position statement” OR “Delphi study” https://www.webofscience.com/wos/woscc/summary/0c78159e-3dc6-4cfb-b66b-e3c82dfdf8c8-0181e71ad4/relevance/1	English; 2015–2025; Consensus development, Delphi study, Clinical practice guideline, Systematic review; Gender: women	1,816
Embase	14 Apr. 2025−20 Nov. 2025	(‘physical activity’:ab,ti OR ‘exercise’:ab,ti) AND (‘pregnancy’:ab,ti OR ‘pregnancy planning’:ab,ti OR preconception:ab,ti OR ‘pregnant women’:ab,ti) AND (‘guideline*’:ab,ti OR ‘recommendation*’:ab,ti OR ‘consensus’:ab,ti OR ‘Delphi study’:ab,ti)	English; 2015–2025; Consensus development, Delphi study, Clinical practice guideline, Systematic Review; Gender: women	560

### 2.3 Stage 3: study selection

All retrieved records were imported into Mendeley and Rayyan for organisation and duplicate removal. Screening was conducted in two stages. At the title and abstract stage, one reviewer screened all records, while a second reviewer independently screened a random [20%] sample of records and all records deemed uncertain to verify consistency in the application of the eligibility criteria. At the full-text stage, all potentially eligible articles were assessed independently by two reviewers. Discrepancies were resolved through discussion among the authors team. Reasons for exclusion at the full-text stage were documented in Rayyan software.

### 2.4 Eligibility criteria

We included clinical practice guidelines, expert consensus statements, position papers or recommendations developed by professional societies, working groups or Delphi panels that addressed physical activity in the context of cancer survivorship, pregnancy planning or pregnancy. Exclusion criteria included studies only on male survivors, animal models, interventions unrelated to physical activity, documents not providing explicit recommendations, guidelines funded by a commercial company (e.g., pharmaceutical or device company), complementary and alternative medicine interventions not used in rehabilitation practice (e.g., herbal remedies, essential oils, etc.) or include interventions that are not movement-based or functional activities (e.g., Reiki, energy therapy, etc.), guidelines for managing the psychological impact of cancer treatment (e.g., anxiety, depression, sexual desire), guidelines for pharmacological interventions only with no functional therapeutic recommendations and developed for health conditions other than cancer or cancer-related symptoms and impairments (e.g., cancer prevention guidelines, health and fitness guidelines for general health maintenance). When multiple updated versions of the same guideline or recommendation were identified within the inclusion period, only the most recent version was retained to avoid duplication. Inclusion and exclusion criteria are summarised in Table 4.

**Table 4:** Inclusion and exclusion criteria.

Criterion	Inclusion	Exclusion
Population	Women with history of cancer and/or planning pregnancy or currently pregnant	Male survivors; studies involving only animal models
Concept	Physical activity, exercise or movement-related recommendations (including type, intensity, frequency, duration)	Interventions or recommendations unrelated to physical activity
Context/source type	Clinical practice guidelines, expert consensus statements, position papers or recommendations developed by professional societies, working groups or Delphi panels	Individual research studies, narrative reviews, commentaries or editorials without explicit recommendations, guidelines funded by commercial company
Language	English-language publications	Non-English sources (if translation unavailable)
Publication status	Published and publicly accessible documents	Unpublished, retracted or inaccessible documents
Time frame	Publications from 2015 onwards	Publications published before 2015

### 2.5 Stage 4: data charting

Data extraction was performed using a standardised Excel spreadsheet. Extracted information included author(s), year of publication, issuing organisation, country or region, target population (e.g., cancer survivors, women planning pregnancy, pregnant women) and specific recommendations on physical activity (e.g., frequency, intensity, time, type, precautions, special considerations). Extraction was performed by one reviewer and cross-checked by a second reviewer to ensure completeness and accuracy.

### 2.6 Stage 5: collating, summarising and reporting results

The included sources were thematically organised according to the research questions. Data were synthesised and presented in both tabular and narrative form to map similarities, differences and gaps across guidelines, consensus statements and expert positions. Reporting followed the PRISMA-ScR checklist, and a PRISMA-ScR flow diagram was prepared to illustrate the study selection process.

### 2.7 Stage 6: consultation

Although no formal consultation stage was carried out, the review protocol and findings were discussed among the author team to ensure accuracy and alignment with clinical and research priorities. The research team comprised senior researchers with academic background in midwifery, oncology, exercise physiology and exercise oncology. Some researchers were involved in the creation of guidelines.

## 3 Results

### 3.1 Study selection and characteristics of included sources

**Figure 1:**
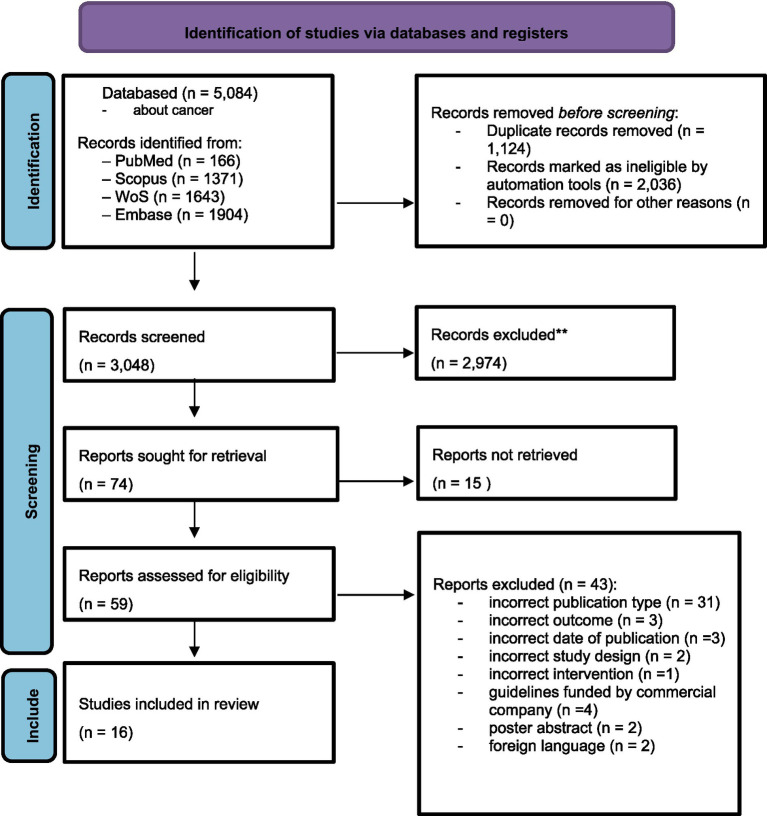
Flow diagram of screening process (studies about recommendations for cancer survivors).

**Figure 2:**
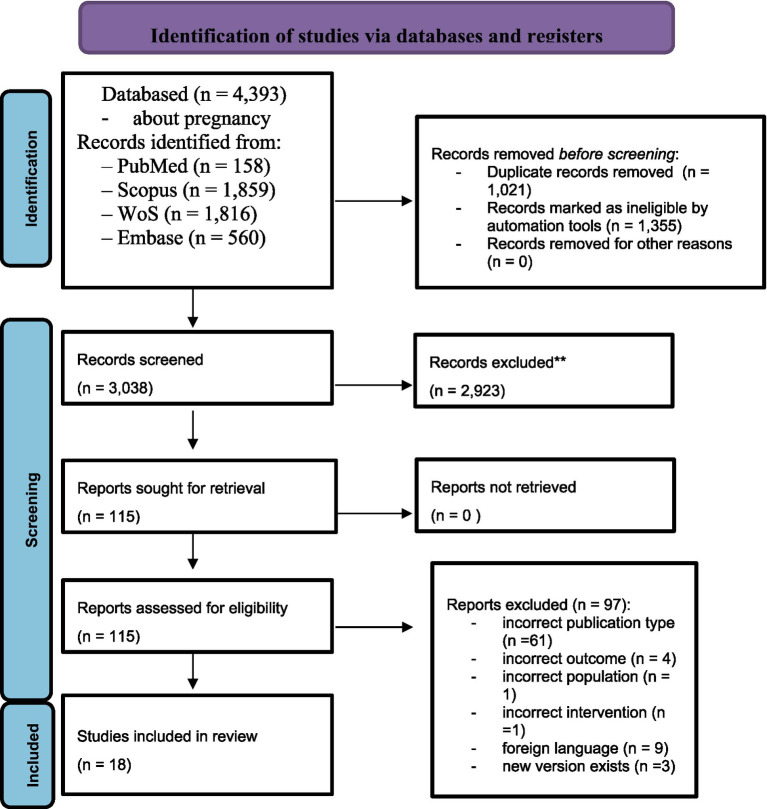
Flow diagram of screening process (studies about recommendations for pregnant women).

In this scoping review, eligible records were identified [*N* = 34] for inclusion (cancer survivorship, *n* = 16; pregnancy/preconception/postpartum, *n* = 18; Figures 1,2). Included sources comprised clinical practice guidelines ([*n* = 19]), professional society statements/position papers ([*n* = 4]), and consensus documents (including Delphi studies; [*n* = 11]). The majority of documents originated from high-income countries and regions. In the cancer survivorship stream (*n* =16), 11/16 sources were produced by organisations based in high-income countries (USA, *n* = 6; Australia, *n =* 1; UK, *n* = 1; Italy, *n* = 1; Japan, *n* = 1; plus one multi-country “Australia/International” panel, *n* = 1), while 4/16 were labelled international and 1/16 did not specify a country/region (CPG working group). In the pregnancy stream (*n* = 18), 10/18 sources originated from high-income countries (USA, *n* = 4; France, *n* = 2; Canada, *n* = 1; Australia, *n* = 1; Poland, *n* = 1; Italy *n* = 1), complemented by Brazil (*n* = 2), international panels (*n* = 3), a regional Asia–Pacific consensus (*n* = 1), and a WHO global consensus output (*n* = 1), reflecting a predominantly high-income evidence-to-guidance society. The publication year distribution of the included sources is presented in (Table 5).

**Table 5:** Publication year distribution.

Year range	Cancer survivorship (*n* = 16)	Pregnancy stream (*n* = 18)
2015–2016	3	3
2017–2018	0	1
2019–2020	3	4
2021–2022	5	6
2023–2025	5	4

For synthesis, sources were organised into two evidence corpora: (1) exercise/physical activity guidance for people living with and beyond cancer (survivorship and rehabilitation), and (2) pregnancy and perinatal physical activity guidance (including preconception and postpartum where available). Although the search strategy included preconception, pregnancy, and postpartum contexts, guidance specifically addressing the preconception period was limited. As a result, the pregnancy-focused corpus was dominated by recommendations for pregnancy, with only limited and less detailed references to preconception and postpartum. No source provided a unified guideline specifically addressing physical activity for women who are cancer survivors and planning pregnancy or pregnant.

The detailed tables summarizing studies conducted in cancer survivors and in pregnancy are provided in Supplementary Tables S1, S2.

### 3.2 Outcome 1: survivorship guidance promotes activity but is less specific for reproductive contexts

Cancer survivorship and oncology rehabilitation guidance consistently endorsed physical activity as a core component of supportive care and long-term health promotion. Recommendations commonly included avoiding inactivity and aiming for doses consistent with adult public health guidance (e.g., 150–300 min per week of moderate-intensity aerobic activity or equivalent, with muscle-strengthening activities on ≥2 days per week) (13, 18–20). Additionally, the ACSM guidelines recommend specific exercise prescriptions for managing outcomes and adverse effects of cancer treatment, where there is moderate to strong evidence of benefits. These include improvements in fatigue, anxiety and depression, quality of life, lymphedema, bone health and sleep quality (18). Some sources provided condition-specific cautions or adaptations relevant to comorbidities or late effects of cancer treatment (e.g., lymphoedema, peripheral neuropathy, treatment-related fatigue, cardiometabolic risk, skeletal fragility or bone metastases) (12, 13, 18, 20–24), but these were not linked to pregnancy physiology or obstetric risk stratification. Overall, survivorship guidance was oriented toward general adult populations rather than women attempting conception or during pregnancy.

### 3.3 Outcome 2: high concordance in core pregnancy physical activity recommendations

Across pregnancy-focused guidelines, there was strong alignment on the minimum recommended dose: ≥150 min per week of moderate-intensity physical activity, typically accumulated over at least 3 days and incorporating both aerobic and resistance modalities (21, 25–28). Pelvic floor muscle training was frequently recommended, alongside advice to minimise prolonged sedentary time (21–23, 25). Most pregnancy guidelines characterised physical activity as safe for uncomplicated pregnancies and emphasised maternal benefits (e.g., reduced risk of gestational diabetes, hypertensive disorders of pregnancy, excessive gestational weight gain, and depressive symptoms), while reporting no increased risk of adverse perinatal outcomes when activity is appropriately prescribed and contraindications are excluded (21, 22, 24, 25, 27). Notably, a comparable safety profile and emphasis on systemic health benefits of physical activity are described in consensus-based oncology guidelines (e.g., ASCO, ACSM) (18, 29).

### 3.4 Outcome 3: central gap-absence of integrated guidance for pregnancy after cancer

Despite a substantial body of guidance for pregnancy physical activity and a parallel body for cancer survivorship, no integrated, population-specific guidance was identified for women who (a) survived cancer and are planning pregnancy, (b) are pregnant after cancer, (c) are pregnant while receiving or having recently completed systemic oncologic treatment (e.g., chemotherapy in the second and third trimesters), or (d) require tailored advice because of prior oncologic treatments (e.g., cardiotoxic therapies, pelvic/abdominal radiotherapy, endocrine therapy or treatment-associated bone loss) (12, 13, 18, 19, 30). The lack of integration was evident in three domains: (1) eligibility/contraindications (obstetric vs. oncology late effects), (2) monitoring and safety thresholds (pregnancy warning signs vs. oncology-related red flags), and (3) service delivery models (clear referral and supervision pathways for a dual-risk population).

### 3.5 Outcome 4: safety framing differs according to corpus-structured in pregnancy guidance, dispersed in oncology guidance

Pregnancy guidance tended to provide explicit contraindications, warning signs to stop exercise and pragmatic modifications (e.g., heat/hydration, trauma/fall risk, position-specific considerations, symptom-triggered adjustments) (21, 22, 25, 27, 31–33). In contrast, oncology guidance more often framed safety through treatment sequelae and comorbidity-based screening, but without translating these into pregnancy-specific risk management or trimester-based physiological considerations (13, 18–20, 29). As a result, applying either set of guidelines in isolation creates operational uncertainty for clinicians advising women who fall at the intersection of both contexts.

### 3.6 Outcome 5: implementation and referral pathways are more developed in oncology consensus literature

Implementation considerations—such as integration of qualified exercise professionals, structured referral practices and attention to access barriers—were more prominent in oncology consensus and Delphi-derived sources (34, 35). Pregnancy guidance allowed to emphasise routine counselling within antenatal care, but less frequently, specified interdisciplinary service models for complex cases. In no source was a pathway explicitly connecting survivorship care with preconception counselling and antenatal management for exercise prescription articulated.

### 3.7 Evidence map summary

The included literature supports a two-axis evidence map: context (preconception/pregnancy/postpartum vs. cancer survivorship) and content domain (FITT prescription, contraindications and monitoring, special populations/late effects and implementation/referral models) (Table 6). The most prominent evidence void lies in the intersection of pregnancy (including preconception) and cancer survivorship, where integrated recommendations and care pathways are absent. Detailed FITT synthesis tables are provided in Supplementary Table S3, while Table 6 summarises the evidence map across populations and recommendation domains.

**Table 6:** Evidence map of physical activity guidance across populations and recommendation domains.

Domain	Pregnancy	Cancer survivorship	Pregnancy after cancer
Frequency	✓✓ ≥3 days/week; often daily movement	✓✓ 3–5 days/week aerobic; 2–3 strength	✗ no dedicated guidance
Intensity	✓✓ moderate; vigorous only if habitually active	✓✓ moderate; individualised	✗ no integrated thresholds
Type of exercise	✓✓ aerobic + resistance + pelvic floor muscle training, flexibility	✓✓ aerobic + resistance + functional	✗ no population-specific guidance
Safety and contraindications	✓✓ explicit obstetric contraindications	✓ treatment-related precautions	✗ not integrated
Late effects and special risks	✓ pregnancy-specific	✓✓ cancer-related late effects	✗ not addressed jointly
Monitoring and progression	✓ pregnancy warning signs	✓ functional and symptom-based	✗ no harmonised approach
Implementation and referral	± antenatal counselling + structured screening/decision support (e.g., Get Active Questionnaire for Pregnancy (41))	✓✓ structured survivorship models	✗ no defined pathways

✓✓ well covered, ✓ partially covered, ±inconsistent/indirect, ✗ not addressed.

To present how safety monitoring is framed differently across guidance corpora, in Supplementary Table S4, a comparison is provided of exercise-related pregnancy warning signs with oncology-related red flags commonly referenced in cancer survivorship and treatment guidelines.

**Figure 3:**
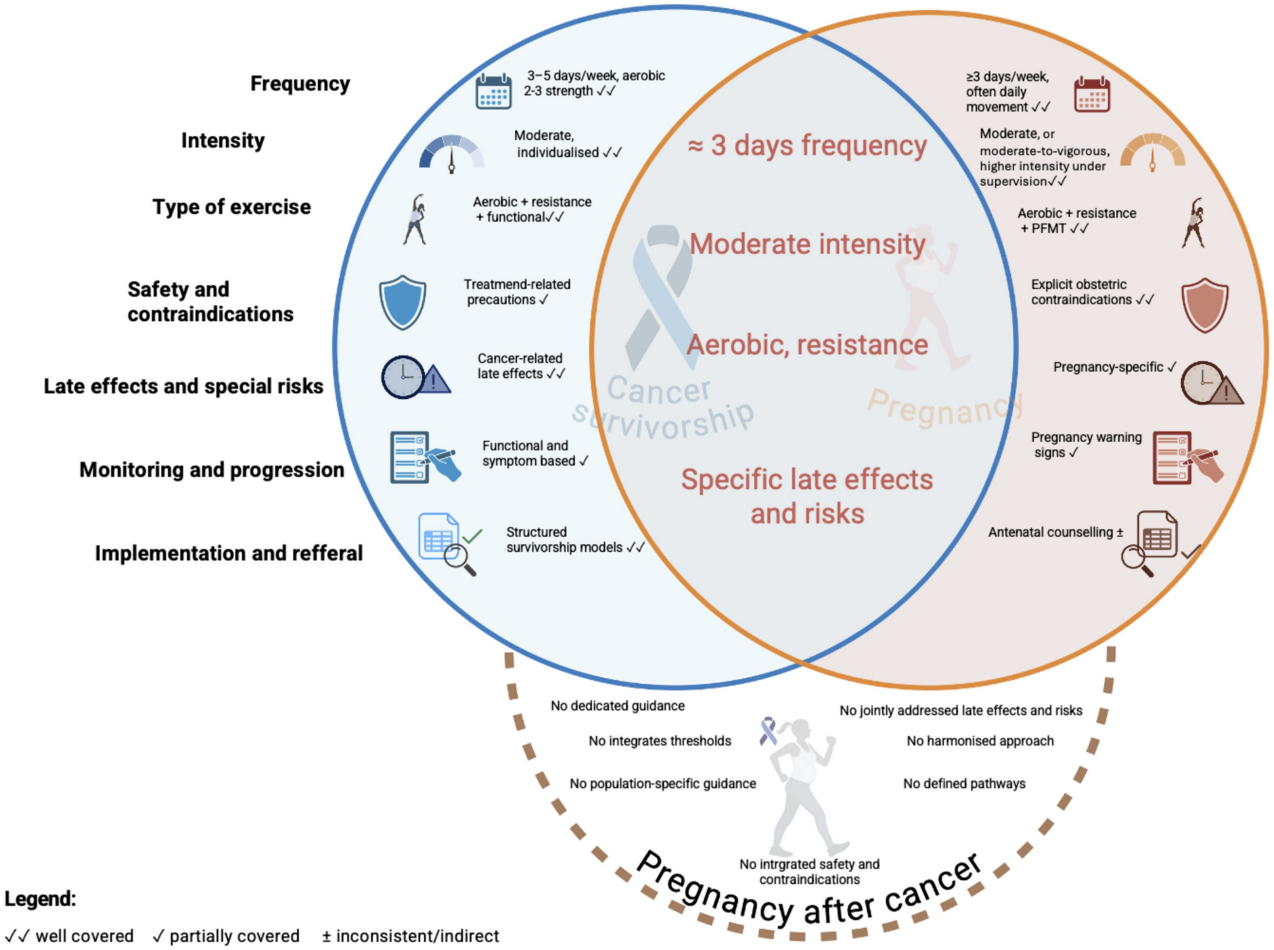
Infographic summary of physical activity recommendations for cancer survivors and women during pregnancy. Created with BioRender.com. Anielska, A. (2026); https://BioRender.com/7in66sn.

The comparison allows to highlight the absence of an integrated set of stop rules and monitoring thresholds for women who are pregnant after cancer or who exercise during or shortly after oncologic treatment. To facilitate comparison across these otherwise siloed guidance corpora, in Figure 3, an infographic summary is given of key physical activity recommendations for women during pregnancy and for cancer survivors.

## 4 Discussion

In this scoping review, physical activity guidance was found to be well established in pregnancy and strongly endorsed in cancer survivorship and rehabilitation literature; however, no integrated, clinically actionable guidance was identified for women who are cancer survivors and are planning pregnancy or are pregnant, and these bodies of guidance remain largely siloed (13, 18–20, 25, 27, 36). Structured contraindications and obstetric safety messaging are offered in pregnancy-related literature, whereas in survivorship literature, the management of treatment sequelae and implementation within survivorship care systems are emphasised. What is missing is a framework reconciling these perspectives into a single, patient-centred approach.

An important finding of this review is that, although pregnancy planning/preconception was included within the review scope, guidance specifically addressing physical activity in the preconception period was sparse. This further underscore the limited development of tailored recommendations for women considering pregnancy after cancer.

These findings are consistent with observations from recent systematic and rapid reviews of oncology rehabilitation and physical activity guidelines, which have highlighted that, despite strong endorsement of physical activity as a standard component of supportive cancer care, existing recommendations are often generic, lack condition-specific adaptation and provide limited operational guidance for complex clinical populations (37–40). In reviews of survivorship guidelines, insufficient integration of comorbidity-specific risk stratification has been noted, as well as late-effect informed exercise modification, and clear implementation pathways across care settings. These issues allow to suggest that the absence of pregnancy-specific survivorship guidance reflects a broader structural limitation in current guideline development rather than an isolated gap identified in this review.

The lack of integrated guidance is clinically consequential because clinicians and patients are currently required to extrapolate from separate pregnancy and cancer survivorship recommendations, often developed in isolation, thereby increasing uncertainty in assessment, risk stratification and exercise prescription in practice. Prior therapies may confer cardiometabolic risk, reduced exercise tolerance, skeletal fragility, lymphatic complications or persistent symptoms such as fatigue and neuropathy. A further challenge is that the included guidance sources rarely defined cancer survivorship according to a consistent temporal framework (e.g., during active treatment, early post-treatment, or long-term survivorship). This limits the extent to which exercise recommendations can be temporally stratified for women planning pregnancy or becoming pregnant after cancer. Pregnancy adds additional physiological demands, symptom fluctuation and obstetric contraindications that can change across trimesters. In this context, simply applying generic pregnancy guidance or generic survivorship guidance may be insufficient to support safe, equitable and effective physical activity participation.

Although dedicated recommendations for pregnancy after cancer were not identified in this review, the overlap between the two corpora suggests a pragmatic starting point: the widely endorsed “minimum dose” of moderate-intensity activity, alongside muscle strengthening, may represent a baseline target for many individuals, provided contraindications are excluded and prescriptions are individualised. However, the operational uncertainty is less about whether activity is advisable and more about how to risk stratify, monitor and adapt intensity as well as modality for women with specific late effects or ongoing survivorship treatments. This underscores the need for multidisciplinary assessment and shared-care models linking oncology, obstetrics and exercise/rehabilitation expertise.

Moreover, contemporary pregnancy guidance increasingly incorporates a patient empowerment and shared decision-making approach, supported by structured self-administered pre-screening tools [e.g., the Get Active Questionnaire for Pregnancy (41)]. Such tools help identify the minority of women who require clinician review before initiating or continuing physical activity, while enabling most women with uncomplicated pregnancies to engage safely and confidently. This model shifts implementation from clinician-led restriction toward risk-informed self-management and supported autonomy—an approach that is largely absent from oncology survivorship guidance, which more often relies on professional referral pathways and clinical risk assessment frameworks. Extending a similar structured, women-centred pre-screening paradigm to cancer survivors planning pregnancy could represent a pragmatic strategy to reduce uncertainty, standardise safety messaging and support equitable access to physical activity counselling.

The predominance of guidance from high-income settings also limits transferability to low- and middle-income countries. In these contexts, the challenge may not be limited to the absence of integrated physical activity guidance alone, but may also reflect more fundamental constraints in access to oncology follow-up, rehabilitation, preconception care, and antenatal services. This suggests that future guidance development should be accompanied by attention to health-system feasibility and context-specific implementation. Implementation challenges are likely to be amplified in routine care, where time constraints, limited cross-disciplinary communication, and variable access to specialised exercise professionals may further hinder translation into practice (42).

The findings support development of a dedicated guidance (e.g., consensus statement or clinical guideline) for physical activity in women planning pregnancy after cancer and during pregnancy after cancer. Priority components include: (1) risk stratification based on oncologic treatment exposures and current comorbidities; (2) minimum screening/assessment elements before initiating or progressing exercise; (3) harmonised stop rules and monitoring parameters combining obstetric warning signs with oncology-related red flags; and (4) clear service delivery pathways and referral criteria. Research priorities include prospective studies reporting maternal, foetal and survivorship outcomes in this population, alongside implementation research evaluating feasible models of integrated care. Addressing this gap represents an important opportunity to advance integrated, patient-centred care across oncology and maternal health.

## 5 Limitations

First of all, included sources were heterogeneous in type and methodological rigour, spanning formal guidelines, position statements, reviews and consensus exercises; scoping review methods map evidence and gaps but do not support comparative effectiveness conclusions. Secondly, the included guidance documents did not consistently define cancer survivorship according to time since treatment or phase of survivorship. As a result, the review could not distinguish clearly between recommendations relevant to women immediately after treatment and those applicable to longer-term survivors. Thirdly, the principal conclusion—a gap in integrated guidance—reflects the absence of dedicated documents and limits the ability to derive population-specific prescriptions for pregnancy after cancer. Fourth, the evidence base is skewed toward high-income settings and English-language guidance, which may under-represent regional practices and resources relevant to low-income and middle-income countries. Fifth, some guidance documents represent updates or variants from the same organisations over time; without careful handling, this may overstate the breadth of independent evidence. In addition, full dual independent screening was not undertaken at the title/abstract stage. Instead, one reviewer screened all records, while a second reviewer independently screened a random subset and all uncertain records; however, all potentially eligible full-text articles were assessed independently by two reviewers. Although this approach was intended to support consistency during the initial screening stage, it may still have increased the risk of missed or inconsistently classified records. Finally, we did not quantify effects or appraise clinical outcomes beyond what was reported within included guidance sources.

## 6 Conclusion

This scoping review demonstrates that largely concordant physical activity recommendations exist for pregnancy and for cancer survivorship when considered independently. However, this guidance leaves a gap for women who have survived cancer and are planning pregnancy or are pregnant. This represents a priority area for future guideline development and interdisciplinary collaboration, with the aim of translating existing evidence into clinically actionable pathways for this growing population. Despite the increasing number of cancer survivors of reproductive age, no dedicated or integrated guidance was identified that would address physical activity prescription across the combined contexts of survivorship and pregnancy. The available literature consistently supports the safety and benefits of physical activity when appropriately prescribed, yet fails to reconcile pregnancy-specific physiological considerations with cancer treatment-related acute and late effects. As a result, clinicians and women are left to extrapolate from population-level recommendations that may not fully capture the complexity of this dual-risk population. The findings underscore a need for integrated, interdisciplinary guidance to support safe, equitable and evidence-informed physical activity counselling for pregnancy after cancer.

## 7 Implications for clinical care

In the absence of integrated guidelines, clinicians supporting women who are cancer survivors and are planning pregnancy or are pregnant should recognise that physical activity is generally beneficial but requires individualised assessment. Existing evidence suggests that population-level minimum recommendations (e.g., ≥150 min per week of moderate-intensity activity with incorporation of muscle strengthening) may serve as a baseline reference but should not be applied uncritically.

Clinical decision-making should incorporate:

- assessment of cancer treatment-related late effects and relevant comorbidities (e.g., cardiotoxicity, skeletal fragility, lymphoedema, persistent fatigue, neuropathy, hypertension, regardless of whether treatment-related, pregnancy-associated or pre-existing);
- evaluation of obstetric status and pregnancy-specific contraindications, which may evolve across gestation;
- ongoing monitoring using both pregnancy warning signs and oncology-related red flags.

Where available, referral to clinicians with expertise in both maternal health and cancer rehabilitation (e.g., physiotherapists or clinical exercise physiologists with oncology experience) may help mitigate risk and support sustained physical activity engagement. The findings highlight the need for interdisciplinary care models linking oncology, obstetrics and exercise or rehabilitation services. Survivorship care plans and preconception counselling represent yet underutilised opportunities to address physical activity proactively. Integrating physical activity assessment and counselling into these pathways may improve care continuity and reduce uncertainty for both clinicians and patients. The absence of integrated recommendations for pregnancy after cancer represents a clear target for future guideline development. From a policy perspective, recognising physical activity as a component of both survivorship care and maternal health may support alignment across traditionally siloed services and contribute to improved long-term outcomes for women and their children.

## Data Availability

Data sharing is not applicable to this article as no new datasets were generated or analysed.
