# Supplementary material for: Guidance on physical activity from cancer survivorship to pregnancy: a scoping review
Source: Front Public Health. 2026 May 29;14:1801672. doi: 10.3389/fpubh.2026.1801672 (PMC13262188; doi:10.3389/fpubh.2026.1801672)
Supplement: Supplementary file 1 [file Supplementary_file_1.docx]

Supplementary Material

# Supplementary Figures and Tables

Table S1. Review of papers presenting studies about cancer survivors

| Author(s)/Year | Year | Issuing organization | Country/Region | Target population | Article type | Specific FITT/PIIT recommendations (frequency, intensity, type) |
| --- | --- | --- | --- | --- | --- | --- |
| Campbell KL et al., 2019 (1) | 2019 | ACSM (American College of Sports Medicine) | International/USA | All adult cancer survivors | Consensus statement/Guidelines | Aerobic: moderate intensity (60-80% HRmax, RPE 13-15), 30-60 min, 3 sessions/week. Resistance: 65-85% 1-RM, 2 sets, 8-12 reps, 2-3 sessions/week. Total goal: 150 minutes/week. |
| Rock CL et al., 2022 (2) | 2022 | American Cancer Society (ACS) | USA | All cancer survivors (adults and children) | Guidelines | Aerobic: 150-300 minutes moderate OR 75-150 minutes vigorous physical activity (PA) per week. Resistance: Muscle-strengthening activities ≥2 days per week [context]. Interventions should be individualised. |
| Runowicz CD et al., 2016 (3) | 2016 | ACS/ASCO | USA | Adult breast cancer survivors | Clinical practice guidelines | Recommends counselling on healthy lifestyle modifications. PA should be offered for musculoskeletal symptoms/pain and neuropathy. No explicit FITT dose provided directly in this guideline excerpt. |
| El-Shami et al., 2015 (4) | 2015 | ACS/ASCO (Colorectal Cancer Survivorship) | USA | Colorectal cancer (CRC) survivors | Clinical practice guidelines | Recommends adherence to general PA/nutrition guidelines. No explicit FITT dose provided directly in this guideline excerpt. |
| Sanft T et al., 2025 (Survivorship, Version 2.2025) (5) | 2025 | National Comprehensive Cancer Network (NCCN) | USA | Adult cancer survivors | Clinical practice guidelines (featured updates) | Time/Intensity: strive for at least 150-300 min/week of moderate-intensity or 75-150 min/week of vigorous-intensity activity (or equivalent combination), spread out over course of week. Type (resistance): engage in 2 to 3 sessions per week of strength/resistance training that include major muscle groups. Type (flexibility/balance): stretch major muscle groups ≥2 days/week; perform core exercises and balance training (especially for older survivors and those at risk of falls). Specifics: avoid prolonged sedentary behaviour. Exercise explicitly recommended to preserve muscle mass when taking weight loss medications (e.g. GLP-1 agonists). |
| Hayes SC et al., 2019 (6) | 2019 | ESSA (Exercise & Sports Science Australia) | Australia | Cancer management (exercise medicine) | Position statement/Guidelines | PA is safe and generally well tolerated during and following treatment. FITT principles are central [context]. No specific FITT dose detailed in provided excerpts. |
| Morrison J et al., 2021 (7) | 2021 | BGCS (British Gynaecological Cancer Society) | UK | Women with uterine cancer | Clinical practice guidelines (guideline) | Regular physical activity reduces risk (Grade A). Recommends early mobilisation in ERAS protocols. No explicit FITT dose. |
| Woopen H et al., 2022 (8) | 2022 | GCIG (Gynecologic Cancer InterGroup) | International | Long-term survivors of gynaecological cancer | Consensus guidelines/Position paper | Patient education on PA helps manage fatigue. Recommends weight-bearing exercise for bone health. No explicit FITT dose. |
| Moraitis AM et al., 2023 (9) | 2023 | International Multidisciplinary Experts | International | Young adult cancer survivors (YACS) (18-39 years) | Expert consensus study (modified Delphi) | Consensus that nature, dose, duration, intensity and frequency (FITT) of PA should be included in prescribed interventions. PA should be integrated during prehabilitation, active treatment and immediate post-treatment phases. |
| Tsuji K. et al., 2025 (10) | 2025 | Japanese Guideline Development Group (MINDS methodology) | Japan | People who have undergone cancer treatment (ages 18-64 and ≥65) | Clinical practice guidelines-type (systematic review + Delphi) | Exercise should be recommended to all physically inactive individuals who have undergone cancer treatment (weak recommendation). No specific FITT details available in sources. |
| Vargo M et al., 2025 (11) | 2025 | CPG Working Group | N/A | Patients with lung cancer | Clinical practice guidelines | Prehabilitation: combined programme of aerobic and other (strengthening/breathing) exercises. During/Post-Tx: combined programme of aerobic, resistance and breathing exercise should be used (Grade B). No specific FITT dose available in sources. |
| Ria Joseph et al., 2023 (12) | 2023 | Expert Panel (funded by Canadian Cancer Society, supported by Cancer Council Queensland) | Australia/International | Cancer survivors (general) | Modified Delphi study (expert consensus) | No specific FITT parameters. Consensus focused on 24 essential elements of referral practices. It recommends that referrals be based on individualised needs in accordance with evidence-based guidelines, but does not define FITT content itself. |
| Pescarenico MG et al., 2021 (13) | 2021 | Italian Association of Medical Oncology (AIOM)/National System for Guidelines (SNLG) | Italy | Long-term cancer survivors (defined as 3-5 years disease-free); lung cancer survivors (stage I-III) | Clinical practice guidelines | Frequency: aerobic: several sessions/week; resistance: 2-3 non-consecutive sessions/week; flexibility: 1–2 times/week. Time/Intensity: ≥150 min/week moderate intensity PA (MPA) OR 75 min/week vigorous intensity PA (VPA), or equivalent combination. Type: combined aerobic and resistance exercise, plus flexibility exercises. Specifics: for lung cancer survivors, low or moderate intensity PA is suggested (conditional recommendation). "Multidimensional" assessment is recommended before prescribing PA. |
| Cohen EEW et al., 2016 (14) | 2016 | American Cancer Society (ACS) | USA | Head and neck cancer (HNC) survivors (post-treatment) | Clinical practice guidelines | Frequency/Time/Intensity: avoid inactivity; aim for at least 150 min/week of moderate or 75 min/week of vigorous aerobic exercise. Type: aerobic exercise plus strength training (≥2 days/week). Specifics: individual rehabilitation referrals recommended for spinal accessory nerve palsy, shoulder dysfunction (range of motion/strength), and trismus (jaw exercises). |
| Clinton SK et al., 2020 (Review of WCRF/AICR Third Expert Report 2018) (15) | 2020 | World Cancer Research Fund (WCRF)/American Institute for Cancer Research (AICR) | International | Cancer survivors (and cancer prevention for general population) | Critical review of expert consensus report | Frequency/Intensity: "be at least moderately physically active and follow or exceed national guidelines". Type: physical activity of all types (occupational, household, transport and recreational). Specifics: limit sedentary habits. Survivors are advised to follow prevention recommendations as much as possible after acute stage of treatment. |
| Ligibel JA et al., 2022 (16) | 2022 | American Society of Clinical Oncology (ASCO) | USA/International | Adults with cancer during active treatment (systemic therapy, radiotherapy, perioperative period) | Clinical practice guidelines (systematic review + expert panel) | Type: aerobic and resistance exercise recommended during active treatment with curative intent. Preoperative exercise may be offered before lung cancer surgery. Frequency/Time: no explicit FITT dose specified; general recommendation to engage in regular exercise consistent with survivorship guidelines. Intensity: not explicitly defined; exercise shown to be safe with low adverse event rates. Specifics: exercise reduces fatigue, preserves cardiorespiratory fitness, muscle strength and physical function; improves QoL and psychological outcomes. Referral to supervised or cancer-specific exercise programmes recommended when clinically indicated. |

Table S2. Review of papers presenting studies about pregnancy

| Author(s)/Year | Year | Issuing organisation | Country/Region | Target population | Article type | Specific FITT/PIIT recommendations (frequency, intensity, type) |
| --- | --- | --- | --- | --- | --- | --- |
| Mottola MF, Davenport MH, et al. (17) | 2019 (2018) | Society of Obstetricians and Gynaecologists of Canada (SOGC) and Canadian Society for Exercise Physiology (CSEP) (Joint SOGC/CSEP Clinical Practice Guideline) | Canada | All women without contraindication should be physically active throughout pregnancy; guidelines also specifically address women who were previously inactive; women diagnosed with gestational diabetes mellitus and categorised as overweight or obese (pre-pregnancy BMI ≥25 kg/m^2^) | Clinical practice guidelines | Time/Duration/Volume: pregnant women should accumulate at least 150 min of moderate-intensity physical activity each week. Frequency (F): activity should be accumulated over a minimum of 3 days per week, though being active every day is encouraged. Intensity (I): moderate-intensity physical activity. Moderate intensity is defined as intense enough to noticeably increase heart rate; a person can talk but not sing during activities of this intensity. Type (T): should incorporate a variety of aerobic exercise and resistance training activities. Adding yoga and/or gentle stretching may also be beneficial. Pelvic floor muscle training (PFMT) (e.g. Kegel exercises) may be performed on a daily basis. |
| Ryan Lee, Serene Thain, et al. IPRAMHO Exercise in Pregnancy Committee (18) | 2021 | Asia-Pacific Consensus (developed by 18 key members representing 10 countries in Asia-Pacific regions) in conjunction with IPRAMHO Exercise in Pregnancy Committee | Asia-Pacific region (countries represented include Singapore, Malaysia, Thailand, Myanmar, India, Indonesia, Vietnam, China, Sri Lanka and Australia) | Pregnant women living in Asia-Pacific regions during the antenatal and postpartum period; applies to all women without contraindications | Expert consensus statement/consensus recommendation | Time/Duration/Volume: accumulate at least 150 min of moderate-intensity physical activity each week. Frequency (F): activity should be accumulated over a minimum of 3 days per week, although being active daily is encouraged. Intensity (I): moderate-intensity (MVPA: 40%-59% heart rate reserve (HRR), typically monitored using the ‘talk test’ (can talk but cannot sing). Type (T): should incorporate a variety of aerobic exercise and resistance training activities. Examples include brisk walking, swimming, stationary cycling, low-impact aerobics, jogging, modified yoga and modified Pilates. Pelvic floor muscle training (PFMT) can be initiated in the immediate postpartum period and may be performed on a daily basis. |
| Rita E Deering, Gráinne M. Donnelly, et al. (19) | 2024 | International Delphi study and consensus statement developed by Clinical and Exercise Professionals (author group represents five different countries) | International (authors represent institutions in USA, UK, Norway, Canada and Australia) | Postpartum runners (females returning to running after childbirth); recommendations are for both elite and recreational runners | International Delphi study and consensus statement | Programme design: programme should be individualised and incorporate a period of relative rest after childbirth. The implementation/dosing of exercise is considered more important than exact time of return. Type (T): initiate training slowly with a walk-run protocol. Incorporate strength training (100% consensus) and cross-training. Targeted strength training should focus on pelvic floor muscles (PFMs), abdominal muscles and lower extremity muscles, particularly hip muscles (extensors, abductors, rotators, hamstrings, quads). Progression: progression should be gradual, avoiding drastic increases in volume. Only one variable (e.g. distance, speed) should be changed at a time. It is recommended that duration/volume be progressed before intensity/speed. Monitoring/Adjustment: progression or regression must be determined by biopsychosocial factors (e.g. musculoskeletal/pelvic symptoms, sleep quality, mental health status, lactation status and energy availability). Runners should be educated to stop running and return to walking if pelvic health symptoms arise during run portion. |
| Wendy J. Brown, Melanie Hayman, et al. (20) | 2022 | Guidelines developed through Australian Government Department of Health funding and expert consultation | Australia (Australian guidelines) | All women without pregnancy complications, who are pregnant or planning a pregnancy, and in postpartum period | Review/guidelines/recommendations (adaptation of recently published international guidelines combined with critical umbrella reviews) | Time/Duration/Volume: accumulate 150 to 300 min of moderate intensity PA, or 75 to 150 min of vigorous PA, or an equivalent combination, each week. Frequency (F): be active on most, preferably all days, every week. Do muscle strengthening activities on at least 2 days each week. Type (T): Incorporate aerobic PA/exercise, muscle strengthening activities, and specifically perform pelvic floor exercises during and after pregnancy. Women should also minimise time spent in prolonged sitting and break up long periods of sitting. Intensity (I): moderate intensity (intense enough to noticeably increase heart rate; able to talk but not sing) or vigorous intensity. |
| Ewa Kwiatkowska, Anna Kajdy, et al. (21) | 2024 | Polish Society of Gynaecologists and Obstetricians (PTGiP) and Polish Society of Sports Medicine (PTMS) | Poland | All women with uncomplicated pregnancies, previously inactive/active, obese women and postpartum women | Recommendations | Time/Duration/Volume: at least 150 min of moderate-intensity PA or 75 min of high-intensity exercise per week. Minimise sedentary behaviour. Frequency (F): accumulated over 3 or more days per week, or 3-5 days per week, even every day (for previously active women). Muscle strengthening: 2-3 non-consecutive days per week. Pelvic floor training: 1 to 7 days per week. Intensity (I): moderate intensity (e.g. able to talk but not sing), or high intensity (for previously active women, with continuous monitoring). Intensity should be monitored using Borg RPE (13-14 ‘somewhat hard’) or the 'talk test'. Type (T): incorporate endurance exercises (aerobic, e.g. brisk walking, swimming, cycling), resistance exercises (weight-bearing/bodyweight exercises), stretching and neuromotor exercise (balance, yoga, Pilates). Pelvic floor muscle training (PFMT) should be included daily. Contact sports of carrying a high risk of abdominal injury, sports entailing a high risk of falling and underwater diving are not recommended. |
| Marlon Harmsworth, Charles Savona, et al. (22) | 2023 | European Board and College of Obstetrics and Gynaecology (EBCOG) | European | Pregnant women (addressing risks related to high-intensity exercise and extreme sports) | Position paper | General/Time: continue regular moderate physical activity. If high-intensity periods are included, they should not be more than 15 minutes, alternating with cool-down periods. Intensity: moderate intensity, avoiding over-exertion. Type (safe): safe examples include brisk walking, swimming, cycling, mild aerobics, yoga, Pilates. Type (avoidance): avoid intense activity in high ambient temperatures. Avoid exercise involving high risk of direct abdominal trauma or significant fall (e.g. gymnastics, competitive cycling). Avoid sports with risks of indirect trauma (sudden accelerations/decelerations). Avoid physical activities associated with reduced oxygenation (e.g. scuba diving, high altitude >1,800 m). Avoid assuming supine position for prolonged period after mid-second trimester. |
| American College of Obstetricians and Gynecologists (ACOG) Committee Opinion (23) | 2020 | American College of Obstetricians and Gynecologists (ACOG) | USA | Women with uncomplicated pregnancies (before, during and after pregnancy) | Committee opinion/guideline | Time/Duration/Volume: at least 20-30 minutes per day. Frequency (F): most or all days of week. Intensity (I): moderate intensity (or vigorous, if habitually engaging in such activity). Type (T): should engage in aerobic and strength-conditioning exercises. |
| American College of Obstetricians and Gynecologists (ACOG) Committee Opinion, American Society for Reproductive Medicine (ASRM) (24) | 2019 | American College of Obstetricians and Gynecologists (ACOG) and American Society for Reproductive Medicine (ASRM) | USA | All patients planning to initiate a pregnancy, women of reproductive age (including those with chronic conditions) | Committee opinion (No. 762)/guidelines | Time/Duration/Volume: at least 30 minutes per day. Accumulate a minimum of 150 minutes of moderate exercise per week. Frequency (F): 5 days a week. Intensity (I): moderate intensity. Type (T): regular physical exercise. |
| Nathalie Boisseau (25) | 2022 | French National College of Midwives (Collège National des Sages-Femmes de France) | France | Women in entire perinatal period: preconception, pregnancy and postpartum | Guidelines/expert consensus review | Time/Duration/Volume (pregnancy): accumulate ≥150 minutes of moderate-intensity physical activity per week. Frequency (F) (pregnancy): at least 3 sessions longer than 30 minutes per week. Muscle strengthening: once or twice a week. Intensity (I) (pregnancy): moderate intensity (able to hold conversation but unable to sing). Type (T) (pregnancy): incorporate aerobic activity (e.g. brisk walking, swimming, stationary bicycling, yoga) and strength-conditioning exercises involving large muscle groups. PIIT (sedentary behaviour): limit sedentary time to ≤7 hours a day, regardless of period (preconception, pregnancy, postpartum). Stand up/move for at least one minute every 30 minutes. |
| Benjamin C. Guinhouya et al. (26) | 2022 | French National College of Midwives (Collège National des Sages-Femmes de France) | France | In document, addressed are effects of maternal physical activity during pregnancy on foetal, newborn and child healt;. recommendations cover interventions during perinatal period | Guidelines/review based on methodical synthesis of existing literature | Pregnancy: pregnant women should accumulate ≥150 minutes of moderate-intensity physical activity per week, distributed over at least 3 sessions of duration longer than 30 minutes (grade A). They should also begin or continue strength training involving large muscle groups once or twice a week (grade A). Sedentary time must be limited to ≤7 hours a day (expert consensus). Note: these recommendations align with those presented in overall guidelines for perinatal period. |
| Loretta DiPietro et al. (27) | 2020 | WHO Guideline Development Group (GDG) | Global (WHO) | Pregnant and postpartum women (specifically addressed among other population sub-groups) | Expert consensus/recommendations for future research | Research recommendations (focus on FITT/PIIT gaps): Intensity (I): conduct studies on effects of vigorous-intensity physical activity (before and during pregnancy) on maternal and foetal outcomes. Type/Volume (T/V): investigate effects of various types, intensities, and volumes of regular physical activity on quality of life, sleep, and symptoms of anxiety and depression during pregnancy and postpartum period. PIIT (sedentary behaviour): conduct RCTs on health benefits of breaking up sedentary time with bouts of light-intensity activity. Conduct observational research on joint association of physical activity and sedentary time with maternal and foetal outcomes. Timing/Domain: determine whether timing (before, during or following pregnancy) or specific domains/settings (e.g. leisure-time versus occupational activity) of physical activity affect maternal and foetal outcomes (e.g. pre-term birth, preeclampsia) differentially. |
| Shefali Mathur Christopher et al. (28) | 2023/2024 | International Delphi Study/Consensus Statement (Involving multidisciplinary professionals, including physiotherapists, physicians, and coaches) | International | Postpartum women who run, aiming for safe return-to-running | International Delphi study and consensus statement | Timing (readiness): timeline to initiate running is person-specific but consensus recommends minimum period of 3 weeks of rest and recovery after childbirth. Any acute birth injuries should be completely healed. Pre-requisites (load/impact screening): before starting to run, runner must successfully complete specific load and impact screening tasks without musculoskeletal or pelvic health symptoms (e.g. 30 minutes of walking, 10 single leg squats, 10 repetitions of hopping in place per leg). Progression (type/frequency): initiate running slowly with walk-run protocol. Incorporate strength training into programme. When progressing, only one variable (distance, speed, incline) should be changed at time. Include rest day between runs. Type (strengthening): include strengthening exercises targeting PFM, abdominal muscles and lower extremity muscles (e.g. hip abductors, extensors). PFM training may be performed daily. Adjustment (biopsychosocial factors): training must be regressed (e.g. decrease running volume) if pelvic health symptoms (incontinence, vaginal heaviness), pain, poor sleep or mental health concerns arise. |
| Maria Cecília Marinho Tenório et al. (29) | 2022 | Brazilian Ministry of Health | Brazil | Pregnant and postpartum women (specific working group established) | Development and methods report (for national guidelines) | General scope of guidelines: resulting national guidelines include recommendations regarding amount (frequency, intensity and duration) of PA. The overall goal is to promote adherence to active lifestyle and overcome PA barriers. FITT/PIIT details: this specific document does not provide concrete FITT metrics (e.g. 150 minutes/week) but confirms that final guidelines cover volume, type, and associated risks of physical activity for this specific population group. |
| Gregore I Mielke et al. (30) | 2021 | Brazilian Ministry of Health | Brazil | Pregnant and postpartum women | National guidelines/recommendations | Volume (T): pregnant women should be encouraged to do at least 150 minutes a week of moderate-intensity PA, up to 300 minutes. If previously active, women can engage in 75 to 150 minutes of vigorous PA per week. Intensity (I): moderate intensity (for previously inactive women). Moderate to vigorous intensity (for previously active women). Moderate intensity is defined as RPE 5-6 on 0-10 scale, making conversation difficult; Vigorous intensity is RPE 7-8, making speech impossible. Frequency (F): should be regular (spread over different days of week). Type (T): aerobic exercises such as brisk walking, water aerobics, cycling on stationary bicycle or strength training are recommended. Pelvic floor muscle exercises should be performed during and after pregnancy. Safety/Avoidance: avoid activities with risk of falling or intense physical contact (e.g. football, wrestling). Avoid activities involving excessive heat or high humidity. Supine position should be considered after the 20th week due to risk of vena cava compression. |
| Katrina L. Piercy et al. (31) | 2018 | U.S. Department of Health and Human Services (HHS) | USA | Women during pregnancy and postpartum period | National guidelines (Physical Activity Guidelines for Americans, 2^nd^ edition). | Time/Volume (T/V): women should do at least 150 minutes (2 hours and 30 minutes) of moderate-intensity aerobic activity a week. Frequency (F): aerobic activity should preferably be spread throughout the week. Intensity (I): moderate intensity is recommended. Women who habitually engaged in vigorous-intensity aerobic activity before pregnancy can continue these activities. Type (T): primarily aerobic activity. General guidelines for adults also include muscle-strengthening activities. PIIT (sedentary behaviour): women should move more and sit less throughout day. Activities of any duration count towards weekly total. |
| Sheri R. Colberg et al. (32) | 2016 | American Diabetes Association (ADA) | USA | Pregnant women with or at risk for gestational diabetes mellitus (GDM) or pre-existing diabetes | Position statement | General: women with preexisting diabetes should be advised to engage in regular PA prior to and during pregnancy. Frequency (F): moderate-intensity exercise on most or all days of week. Intensity (I): moderate intensity (20-30 min sessions) is recommended. Vigorous-intensity exercise may be considered for overweight or obese pregnant women with GDM to reduce excess GWG. Time/Duration (T/volume): 20-30 minutes per session. Type (T): both aerobic or resistance training can be used to improve insulin action and glycaemic control in GDM. Precautions (GDM/insulin users): women using insulin should be aware of increased risk of hypoglycaemia due to insulin-sensitising effects of exercise. To prevent hypoglycaemia, specific adjustments to insulin and/or carbohydrate intake are suggested based on pre-exercise blood glucose levels (e.g. ingesting 15-30 g of fast-acting carbohydrate if blood glucose is <90 mg/dL). |
| Giuseppe Musumeci (33) | 2016 | Italian Ministry of Health (via commentary/overview) | Italy | Pregnant women | Editorial/summary of guidelines | Duration/Volume (T): 30-40 minutes a day. Intensity (I): low intensity. Type (T): walking, swimming or gymnastics. Modification/Progression: after the eighth month of pregnancy, only perform exercises to improve breathing and relaxation. Exercise regimens must be mild to moderate and personalised ("tailor-made"). |
| Bø et al. (34) | 2016 | IOC Expert Group Meeting (International Olympic Committee) | International | High-level regular exercisers and elite athletes during pregnancy | Expert consensus/evidence summary | Intensity (I): maximal VO2 testing and exercise above 90% of maximal maternal HR is NOT recommended. Target HR ranges for training based on fitness: 145-160 bpm (age 20-29) and 140-156 bpm (age 30-39). The Borg RPE scale should not be used as the only measure of exercise intensity from second trimester, as it may underestimate HR. Type (T): endurance training can be maintained. Light-to-moderate weight training (resistance training) is generally acceptable. Heavy strength training should be performed with caution or avoided, particularly due to the Valsalva manoeuvre (which may temporarily decrease foetal blood flow) and potential harm to pelvic floor. Safety/Avoidance: avoid sports that pose a high risk of direct abdominal trauma or significant falls (e.g. horseback riding, ice skating, downhill skiing, combat sports). Avoid high-intensity training regimes at altitudes greater than 1,500-2,000 m. Scuba diving is prohibited due to the risk of foetal decompression sickness. Position/PIIT: avoid supine position for prolonged periods after mid-second trimester to prevent compression of inferior *vena cava*. Ensure adequate hydration and avoid exercise in high heat or humidity to mitigate hyperthermia risk. |

**Table S3** Summary of physical activity recommendations for cancer survivors, grouped according to ‘frequency’, ‘intensity’ and ‘type’ (FITT framework)

**Frequency**

| **Recommendation** | **Sources (examples)** | **Notes** |
| --- | --- | --- |
| ≥3 days per week | ACSM (1); ACS (2); NCCN Survivorship (5); AIOM (13) | Most common minimum frequency |
| 3-5 days per week | ESSA (6); ACSM (1); ACS (2) | Often for aerobic exercise |
| 2-3 days per week (strength) | ACSM (1); ACS (2); NCCN (5) | For major muscle groups |
| Avoid ≥2 consecutive inactive days | ESSA (2019) (6) | Emphasised to prevent deconditioning |
| Gradual progression over weeks | ACSM (1); Delphi consensus papers (9) | Especially post-treatment |

**Intensity**

| **Recommendation** | **Sources (examples)** | **Notes** |
| --- | --- | --- |
| Moderate intensity | ACSM (1); ACS (2); AIOM (13); ESSA (6) | Core recommendation across guidelines |
| Vigorous intensity permitted if tolerated | ACSM (1); ACS(2); NCCN | For selected survivors with supervision |
| Individualised intensity prescription | ACSM (1); NCCN (5); Delphi studies | Based on treatment side effects |
| Use RPE / talk test / HRmax | ACSM (1) | HRR may overestimate intensity in survivors |
| Avoid maximal intensity initially | Multiple guidelines (1,4,5,7,8,14) | Especially with cardiotoxicity/fatigue |

**Type**

| **Type of activity** | **Sources (examples)** | **Notes** |
| --- | --- | --- |
| Aerobic exercise | All survivorship guidelines | Walking, cycling, swimming most common |
| Resistance/strength training | ACSM (1); ACS (2); ESSA (6); NCCN (5) | Safe, incl. lymphoedema, mainly for breast cancer (progressive) |
| Combined aerobic + resistance | ACSM (1); ACS (2); AIOM (13) | Best for quality of life ( QoL) and fatigue |
| Balance and functional training | NCCN (5); ACS (2) | Especially older survivors and those who were exposed to neurotoxic chemotherapy (taxane and platinum-based) (2) |
| Flexibility/stretching | ACSM (2); NCCN (5) | Adjunct, not standalone |
| Condition-specific modifications | ACSM; lymphoedema guidelines (1) | Load management critical for treatment-related conditions (e.g. lymphoedema, neuropathy and treatment-associated bone disorders) |

When mapped using the FITT framework, cancer survivorship guidelines consistently recommend regular aerobic and resistance exercise at moderate intensity, strongly emphasizing individualisation based on treatment-related late effects and functional capacity.

Table S2. Exercise-related pregnancy warning signs versus oncology-related red flags relevant to physical activity prescription

| **Domain** | **Pregnancy: warning signs to stop exercise** | **Cancer survivorship/active treatment: oncology-related red flags** |
| --- | --- | --- |
| **Cardiorespiratory symptoms** | Chest pain; persistent or worsening shortness of breath not resolving with rest | Chest pain; syncope or near-syncope; hypotension; new or worsening dyspnoea during or after exercise |
| **Neurological/systemic symptoms** | Dizziness or faintness; severe or persistent headache | Dizziness; syncope; marked intolerance to usual exercise intensity; acute neurological symptoms |
| **Obstetric-specific symptoms** | Vaginal bleeding; painful or regular uterine contractions; persistent leakage of fluid (suspected rupture of membranes) | — |
| **Musculoskeletal/pain-related concerns** | Exercise modification guided by tolerance and obstetric contraindications | New or worsening musculoskeletal pain; suspected skeletal fragility; pain suggestive of bone metastases; loss of balance or coordination |
| **Treatment or condition-related risks** | Conditions requiring caution or restriction (e.g. severe hypertensive disorders of pregnancy, symptomatic anaemia, selected cardiac or respiratory conditions) | Treatment-related late or acute effects requiring modification (e.g. cardiotoxicity, peripheral neuropathy, lymphoedema, treatment-related bone disease); need for pre-exercise risk stratification |
| **Recommended action** | Stop exercise and seek obstetric evaluation | Stop or modify exercise and seek oncology/rehabilitation review; individualised reassessment before resuming |

**Note**: Pregnancy guidelines typically present explicit, standardised warning signs prompting cessation of exercise, whereas oncology guidelines frame safety primarily through treatment-related risk assessment, symptom monitoring and low reported rates of serious adverse events. No guideline integrates these safety frameworks for women who are pregnant after cancer or pregnant during/after systemic oncologic treatment.

References:

1. Campbell KL, Winters-Stone KM, Wiskemann J, May AM, Schwartz AL, Courneya KS, Zucker DS, Matthews CE, Ligibel JA, Gerber LH, et al. Exercise Guidelines for Cancer Survivors: Consensus Statement from International Multidisciplinary Roundtable. *Med Sci Sports Exerc* (2019) 51:2375–2390. doi: 10.1249/MSS.0000000000002116

2. Rock CL, Thomson CA, Sullivan KR, Howe CL, Kushi LH, Caan BJ, Neuhouser ML, Bandera E V., Wang Y, Robien K, et al. American Cancer Society nutrition and physical activity guideline for cancer survivors. *CA Cancer J Clin* (2022) 72:230–262. doi: 10.3322/CAAC.21719

3. Runowicz CD, Leach CR, Henry NL, Henry KS, Mackey HT, Cowens-Alvarado RL, Cannady RS, Pratt-Chapman ML, Edge SB, Jacobs LA, et al. American Cancer Society/American Society of Clinical Oncology Breast Cancer Survivorship Care Guideline. *CA Cancer J Clin* (2016) 66:43–73. doi: 10.3322/caac.21319

4. El-Shami K, Oeffinger KC, Erb NL, Willis A, Bretsch JK, Pratt-Chapman ML, Cannady RS, Wong SL, Rose J, Barbour AL, et al. American Cancer Society Colorectal Cancer Survivorship Care Guidelines. *CA Cancer J Clin* (2015) 65:428–455. doi: 10.3322/caac.21286

5. Sanft T, Day AT, Ansbaugh SM, Ariza-Heredia EJ, Armenian S, Baker KS, Ballinger TJ, Cathcart-Rake EJ, Cohen SH, Evgeniou E, et al. NCCN Guidelines® Insights: Survivorship, Version 2.2025. *J Natl Compr Canc Netw* (2025) 23:208–217. doi: 10.6004/jnccn.2025.0028

6. Hayes SC, Newton RU, Spence RR, Galvão DA. The Exercise and Sports Science Australia position statement: Exercise medicine in cancer management. *J Sci Med Sport* (2019) 22:1175–1199. doi: 10.1016/j.jsams.2019.05.003

7. Morrison J, Balega J, Buckley L, Clamp A, Crosbie E, Drew Y, Durrant L, Forrest J, Fotopoulou C, Gajjar K, et al. British Gynaecological Cancer Society (BGCS) uterine cancer guidelines: Recommendations for practice. *Eur J Obstet Gynecol Reprod Biol* (2022) 270:50–89. doi: 10.1016/j.ejogrb.2021.11.423

8. Woopen H, Sehouli J, Davis A, Lee YC, Cohen PA, Ferrero A, Gleeson N, Jhingran A, Kajimoto Y, Mayadev J, et al. GCIG-Consensus guideline for Long-term survivorship in gynecologic Cancer : A position paper from the gynecologic cancer Intergroup ( GCIG ) symptom benefit committee. *Cancer Treat Rev* (2022) 107:102396. doi: 10.1016/j.ctrv.2022.102396

9. Moraitis AM, Seven M, Sirard J, Walker R. Expert Consensus on Physical Activity Use for Young Adult Cancer Survivors’ Biopsychosocial Health: A Modified Delphi Study. *J Adolesc Young Adult Oncol* (2022) 11:459–469. doi: 10.1089/jayao.2021.0109

10. Tsuji K, Sasai H, Kiyohara K, Nakata Y, Nishiwaki H, Ohta T, Ochi E, Takano T, Tatematsu N, Matsuoka YJ. Japan’s cancer survivorship guidelines for exercise and physical activity. *Jpn J Clin Oncol* (2025) 55:12–20. doi: 10.1093/jjco/hyae126

11. Vargo M, Gerber LH, Gilchrist LS, Fisher MI. Recommendations for Interventions to Improve Function in Patients With Lung Cancer: A Clinical Practice Guideline. *Cancer Med* (2025) 14:e70626. doi: 10.1002/cam4.70626

12. Joseph R, Hart NH, Bradford N, Wallen MP, Han CY, Pinkham EP. Essential elements of optimal dietary and exercise referral practices for cancer survivors : expert consensus for medical and nursing health professionals. *Support Care Cancer* (2023)1–14. doi: 10.1007/s00520-022-07509-1

13. Pescarenico MG, Artioli F, Beretta G, Bonotto M, Caraceni AT, Carnio S, Ferraù F, Giusti R, Micallo G, Numico G, et al. Physical activity and long-term cancer survivors: Italian Association of Medical Oncology (AIOM)/SNLG guidelines 2020. *J Cancer Rehabil* (2021) 4:60–70. doi: 10.48252/JCR18

14. Cohen EEW, LaMonte SJ, Erb NL, Beckman KL, Sadeghi N, Hutcheson KA, Stubblefield MD, Abbott DM, Fisher PS, Stein KD. American Cancer Society head and neck cancer survivorship care guideline. *CA Cancer J Clin* (2016) 66:203–239.

15. Clinton SK, Giovannucci EL, Hursting SD. The World Cancer Research Fund/American Institute for Cancer Research Third Expert Report on Diet, Nutrition, Physical Activity, and Cancer: Impact and Future Directions. *J Nutr* (2020) 150:663–671. doi: https://doi.org/10.1093/jn/nxz268

16. Ligibel JA, Bohlke K, May AM, Clinton SK, Demark-Wahnefried W, Gilchrist SC, Irwin ML, Late M, Mansfield S, Marshall TF, et al. Exercise, Diet, and Weight Management During Cancer Treatment: ASCO Guideline. *J Clin Oncol* (2022) 40:2491–2507. doi: 10.1200/JCO.22.00687

17. Mottola MF, Davenport MH, Ruchat S-M, Davies GA, Poitras VJ, Gray CE, Jaramillo Garcia A, Barrowman N, Adamo KB, Duggan M, et al. 2019 Canadian guideline for physical activity throughout pregnancy. *Br J Sports Med* (2018) 52:1339–1346. doi: 10.1136/bjsports-2018-100056

18. Lee R, Thain S, Tan LK, Teo T, Tan KH. Asia-Pacific consensus on physical activity and exercise in pregnancy and the postpartum period. *BMJ open Sport Exerc Med* (2021) 7:e000967. doi: 10.1136/bmjsem-2020-000967

19. Deering RE, Donnelly GM, Brockwell E, Bo K, Davenport MH, De Vivo M, Dufour S, Forner L, Mills H, Moore IS, et al. Clinical and exercise professional opinion on designing a postpartum return-to-running training programme: an international Delphi study and consensus statement. *Br J Sports Med* (2024) 58:183–195. doi: 10.1136/bjsports-2023-107490

20. Brown WJ, Hayman M, Haakstad LAH, Lamerton T, Mena GP, Green A, Keating SE, Gomes GAO, Coombes JS, Mielke GI. Australian guidelines for physical activity in pregnancy and postpartum. *J Sci Med Sport* (2022) 25:511–519. doi: 10.1016/j.jsams.2022.03.008

21. Kwiatkowska E, Kajdy A, Sikora-Szubert A, Karowicz-Bilinska A, Zembron-Lacny A, Ciechanowski K, Krzywanski J, Kwiatkowski S, Kostka T, Sieroszewski P, et al. Polish Society of Gynecologists and Obstetricians (PTGiP) and Polish Society of Sports Medicine (PTMS) recommendations on physical activity during pregnancy and the postpartum period. *Ginekol Pol* (2024) 95:218–231. doi: {}

22. Harmsworth M, Savona-Ventura C, Mahmood T. High-intensity exercise during pregnancy - A position paper by the European Board and College of Obstetrics and Gynaecology (EBCOG). *Eur J Obstet Gynecol Reprod Biol* (2023) 285:56–58. doi: 10.1016/j.ejogrb.2023.03.038

23. American College of Obstetricians and Gynecologists. Physical activity and exercise during pregnancy and the postpartum period. *Obstet Gynecol* (2020) 135:e178–e188.

24. American College of Obstetricians and Gynecologists. ACOG Committee Opinion No. 762: Prepregnancy Counseling. *Obstet Gynecol* (2019) 133:e78–e89. doi: 10.1097/AOG.0000000000003013

25. Boisseau N. Physical Activity During the Perinatal Period: Guidelines for Interventions During the Perinatal Period from the French National College of Midwives. *J Midwifery Womens Health* (2022) 67 Suppl 1:S158–S171. doi: 10.1111/jmwh.13425

26. Guinhouya BC, Duclos M, Enea C, Storme L. Beneficial Effects of Maternal Physical Activity during Pregnancy on Fetal , Newborn , and Child Health : Guidelines for Interventions during the Perinatal Period from the French National College of Midwives. *J Midwifery Womens Health* (2022)149–157. doi: 10.1111/jmwh.13424

27. DiPietro L, Al-Ansari SS, Biddle SJH, Borodulin K, Bull FC, Buman MP, Cardon G, Carty C, Chaput J-P, Chastin S, et al. Advancing the global physical activity agenda: recommendations for future research by the 2020 WHO physical activity and sedentary behavior guidelines development group. *Int J Behav Nutr Phys Act* (2020) 17:143. doi: 10.1186/s12966-020-01042-2

28. Christopher SM, Donnelly G, Brockwell E, Bo K, Davenport MH, De Vivo M, Dufour S, Forner L, Mills H, Moore IS, et al. Clinical and exercise professional opinion of return-to-running readiness after childbirth: an international Delphi study and consensus statement. *Br J Sports Med* (2024) 58:299 LP – 312. doi: 10.1136/bjsports-2023-107489

29. Marinho Tenório MC, Coelho-Ravagnani C, Umpierre D, Andrade DR, Autran R, de Barros MVG, Benedetti TRB, Cavalcante FVSA, Cyrino ES, Dumith SC, et al. Physical Activity Guidelines for the Brazilian Population: Development and Methods. *J Phys Act Health* (2022) 19:367–373. doi: 10.1123/jpah.2021-0756

30. Mielke GI, Tomicki C, Botton CE, Cavalcante FVSA, Borges GF, Galliano LM, Sandreschi PF, Pinto SS, Bezerra TA, Hallal PC, et al. Physical activity for pregnant and postpartum women: Physical Activity Guidelines for the Brazilian Population. *Rev Bras Atividade Física Saúde* (2021) 26:e0217. doi: 10.12820/rbafs.26e0217

31. Piercy KL, Troiano RP, Ballard RM, Carlson SA, Fulton JE, Galuska DA, George SM, Olson RD. The Physical Activity Guidelines for Americans. *JAMA* (2018) 320:2020–2028. doi: 10.1001/jama.2018.14854

32. Colberg SR, Sigal RJ, Yardley JE, Riddell MC, Dunstan DW, Dempsey PC, Horton ES, Castorino K, Tate DF. Physical Activity/Exercise and Diabetes: A Position Statement of the American Diabetes Association. *Diabetes Care* (2016) 39:2065–2079. doi: 10.2337/dc16-1728

33. Musumeci G. Physical Activity for Health—An Overview and an Update of the Physical Activity Guidelines of the Italian Ministry of Health. *J Funct Morphol Kinesiol* (2016) 1:269–275. doi: 10.3390/jfmk1030269

34. Bø K, Artal R, Barakat R, Brown W, Davies GAL, Dooley M, Evenson KR, Haakstad LAH, Henriksson-Larsen K, Kayser B, et al. Exercise and pregnancy in recreational and elite athletes: 2016 evidence summary from the IOC expert group meeting, Lausanne. Part 1—exercise in women planning pregnancy and those who are pregnant. *Br J Sports Med* (2016) 50:571–589. doi: 10.1136/bjsports-2016-096218
